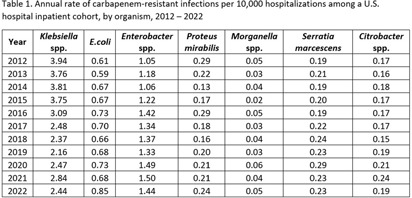# Organism-specific Trends in Carbapenem-resistant Enterobacterales Infections in a Cohort of Hospitalized Patients, 2012–2022

**DOI:** 10.1017/ash.2024.337

**Published:** 2024-09-16

**Authors:** Mohammed Khan, Hannah Wolford, Natalie McCarthy, Babatunde Olubajo, Jonathan Bishop, James Baggs, Joseph Lutgring, Sujan Reddy, Maroya Walters

**Affiliations:** Centers for Disease Control and Prevention

## Abstract

**Background:** Carbapenem-resistant Enterobacterales (CRE) infections are an urgent public health threat. An estimated 12,700 CRE (including E. coli, Klebsiella spp., and Enterobacter spp.) infections occurred in the United States in 2020. While the estimated incidence of CRE infections has been relatively stable between 2012 and 2020, organism-specific trends, including those for organisms not typically included in CRE surveillance definitions, have not been described. We estimated the annual rate of carbapenem-resistant Enterobacterales infections, disaggregated by organism, from 2012 to 2022. **Methods:** Data on inpatient hospitalizations from a dynamic cohort of short-term acute care hospitals reporting microbiology data between 2012 and 2022 were obtained from the PINC AI Database and the BD Insights Research Database. We included patients with clinical isolates of E. coli, Enterobacter spp., Klebsiella spp., Citrobacter spp., Serratia marcescens, Proteus mirabilis, and Morganella spp. and sufficient susceptibility results to identify carbapenem resistance. We limited our analysis to incident isolates, defined as a patient’s first isolate of a given organism and carbapenem resistance phenotype in a 14-day period. We calculated the annual rate of carbapenem-resistant infections per 10,000 hospitalizations for each organism. **Results:** There were 3,018,792 incident isolates from 55.8 million hospitalizations included in the analysis. Overall, 31,226 incident carbapenem-resistant isolates were identified. The rate of carbapenem-resistant infections varied by organism and over time (Table 1). The rate of carbapenem-resistant Klebsiella spp. infections appeared to decline from 3.94 in 2012 to 2.44 infections per 10,000 hospitalizations in 2022. The rate of carbapenem-resistant Enterobacter spp. infections appeared to increase from 1.05 in 2012 to 1.44 infections per 10,000 hospitalizations in 2022. The rate of carbapenem-resistant E. coli infections also appeared to increase, from 0.61 in 2012 to 0.85 infections per 10,000 hospitalizations in 2022. Rates of carbapenem-resistant Proteus mirabilis, Morganella spp., Citrobacter spp., or Serratia marcescens infections were similar in 2022 compared to 2012. **Conclusions:** Disaggregating data by organism revealed heterogeneous trends, with apparent increases in rates of carbapenem-resistant Enterobacter spp. and E. coli infections and apparent decreases in rates of carbapenem-resistant Klebsiella spp. infections. Organism-specific CRE analyses may provide additional insight into CRE epidemiology.

**Figure:**